# Optimization of RiPCA for the Live‐Cell Detection of Pre‐MicroRNA‐Protein Interactions

**DOI:** 10.1002/cbic.202200508

**Published:** 2022-11-22

**Authors:** Sydney L. Rosenblum, Amanda L. Garner

**Affiliations:** ^1^ Department of Medicinal Chemistry University of Michigan Ann Arbor Michigan 48109 USA; ^2^ Program in Chemical Biology University of Michigan Ann Arbor Michigan 48109 USA

**Keywords:** cell-based assays, hnRNP A1, Lin28, Musashi 1/2, pre-microRNA, RiPCA, RNA-binding proteins

## Abstract

Advancements in methods for identifying RNA‐protein interactions (RPIs) on a large scale has necessitated the development of assays for validation of these interactions, particularly in living cells. We previously reported the development of RiPCA (RNA interaction with protein‐mediated complementation assay) to enable the cellular detection of the well‐characterized interaction between the pre‐microRNA, pre‐let‐7, and its RNA‐binding protein (RBP) partner Lin28. In this study, the applicability of RiPCA for the detection of putative pre‐miRNA‐protein interactions was explored using an improved RiPCA protocol, termed RiPCA 2.0. RiPCA 2.0 was adapted to detect the sequence specificity of the RBPs hnRNP A1, Msi1, and Msi2 for reported pre‐microRNA binding partners. Additionally, the ability of RiPCA 2.0 to detect site‐specific binding was explored. Collectively, this work highlights the versatility of RiPCA 2.0 in detecting cellular RPIs.

## Graphical abstract

**Interest in studying** reported, but under‐characterized RNA‐protein interactions (RPI) warrants the development of tools to detect and validate these interactions. RiPCA is a cell‐based assay capable of detecting pre‐miRNA‐protein interactions in a sequence‐specific manner. This study reports the optimization of RiPCA and its application in detecting interactions between the RBPs hnRNP A1, Msi1, and Msi2 and select pre‐miRNA binding partners.

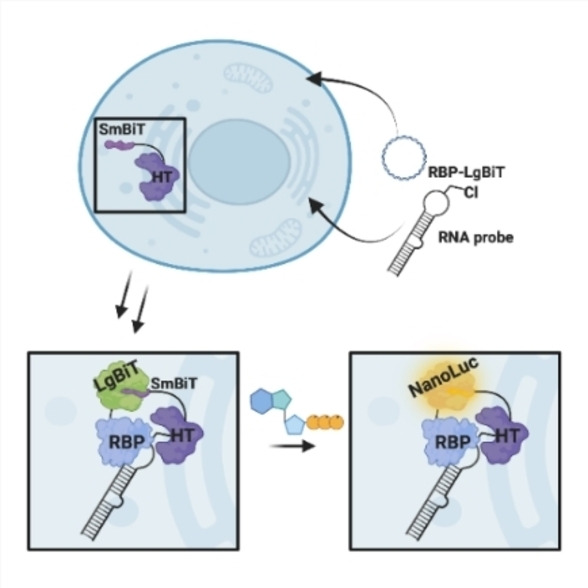

## Introduction

MicroRNAs (miRNAs) are a class of small, non‐coding RNA involved in the post‐transcriptional regulation of >50 % of human protein‐coding genes.^[1]^ Over 2,600 human miRNAs have been identified and found to play a role in the regulation of virtually all cellular processes, including developmental timing, cell proliferation and differentiation, and apoptosis.^[2]^ Consequently, miRNA levels are tied to cellular homeostasis, and thus, aberrant miRNA expression and abundance have been linked to many human diseases including cancers.^[3]^ MiRNAs are generated through a series of processing steps beginning from a long primary transcript (pri‐miRNA), to a shorter precursor miRNA (pre‐miRNA) hairpin loop, and finally to its mature ∼22 nucleotide form.^[4]^ These steps are carried out by RNase III enzymes, Drosha and Dicer, respectively. In addition, miRNA biogenesis can be enhanced or inhibited by auxiliary RNA‐binding proteins (RBPs).[^3a^, ^4^]

The RNA‐protein interaction (RPI) between the let‐7 family of miRNA and the Lin28 RBPs is one of the best characterized examples of RBP regulation of miRNA maturation.^[5]^ Let‐7s are known tumor suppressors that downregulate the translation of prominent oncogenes, such as MYC, RAS, and HMGA2.^[6]^ Lin28 binds to the terminal loop of the let‐7 pri‐ or precursor hairpin and acts as a negative regulator by either physically inhibiting Drosha or Dicer processing or triggering degradation through the addition of an oligouridine tail by terminal uridyltransferases (TUTases).^[7]^ Lin28 expression results in a loss of let‐7 and subsequent de‐repression of oncogenic transcripts, and is frequently elevated in cancers, namely lung and ovarian cancer, hepatocellular carcinoma, and melanoma.[^6^, ^8^] Therefore, the let‐7/Lin28 RPI has garnered attention as a potential target for the development of novel anti‐cancer therapeutics.^[9]^

While the Lin28 proteins are by no means the only RBPs to influence miRNA processing, much less is known about additional regulators.^[3a]^ Much of our knowledge regarding post‐transcriptional regulation of miRNA biogenesis by RBPs has been generated via large‐scale proteomics and sequencing efforts. In an effort to uncover RBPs involved in miRNA maturation, Treiber *et al*. utilized a large‐scale, genome‐wide pull‐down‐proteomics‐based approach to identify proteins bound to 72 pre‐miRNA sequences.^[10]^ From these efforts, ∼180 RBPs were identified across 11 cell lines, binding to a unique subset of pre‐miRNA baits. Taking an RBP‐centric approach, Nussbacher and Yeo mined publicly available enhanced UV crosslinking followed by immunoprecipitation (eCLIP) datasets to identify RBPs enriched for binding at annotated pre‐miRNA loci, revealing 116 such RBPs in HepG2 and K562 cells.^[11]^ As these methods provided an overlapping, yet non‐redundant set of putative pre‐miRNA‐protein interactions,^[11]^ there remains a need for the development of orthogonal technologies for the experimental validation of the interactions identified, particularly those that can be performed in live cells due to the known challenges of *in vitro* methods in studying RPIs.^[12]^

**Figure 1:**
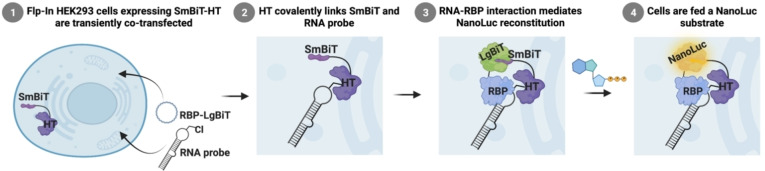
RiPCA scheme. Created with BioRender.com.

Recently, the Garner laboratory developed a cell‐based assay for the direct detection of RPIs called RNA interaction with Protein‐mediated Complementation Assay, or RiPCA (Figure 1).^[13]^ In RiPCA, Flp‐In™ HEK293 cells stably expressing the small subunit of split NanoLuciferase (NanoLuc)^[14]^ (SmBiT) fused to HaloTag^[15]^ (HT) (SmBiT‐HT), either in the cytoplasm or nucleus, are transiently co‐transfected with a plasmid encoding an RBP tagged with the large subunit of NanoLuc (LgBiT) and an RNA probe modified to contain a HT ligand (step 1). Once inside the cell, the RNA probe becomes covalently modified with SmBiT via HT (step 2), and subsequent interactions between the LgBiT‐tagged RBP and SmBiT‐labeled RNA (step 3) are detectable via chemiluminescence following treatment with NanoLuc substrate (step 4). Using the let‐7/Lin28 RPI as proof‐of‐concept, RiPCA was shown to detect sequence‐specific binding to Lin28 in the cytoplasm and nucleus.^[13a]^

Given the modular nature of RiPCA, we sought to demonstrate its applicability for detecting putative pre‐miRNA‐protein interactions identified via proteomics. Herein we report an improved RiPCA protocol, RiPCA 2.0, and describe its application for the detection of pre‐miRNA interactions with heterogeneous nuclear ribonucleoprotein A1 (hnRNP A1) and the Musashi proteins (Msi1 and Msi2). Successful assay development for these RPIs subsequently allowed for the assessment of sequence selectivity against a small library of pre‐miRNA sequences. This additional development and application of the RiPCA technology further demonstrates its utility in studying RPIs in living cells.

## Results and Discussion

### Development of RiPCA 2.0

**Figure 2:**
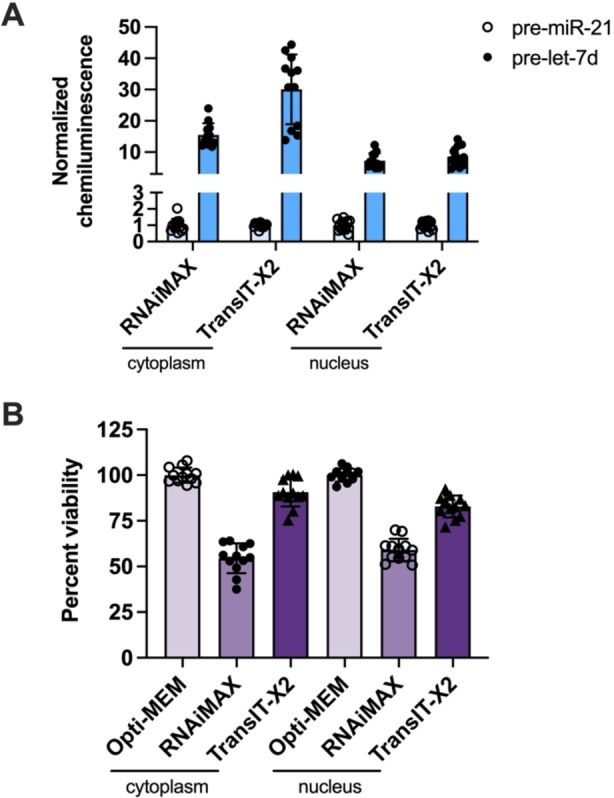
Optimization of transfection protocol leading to the development of RiPCA 2.0. (A) Comparison of Lipofectamine™ RNAiMAX and TransIT‐X2® transfection reagents in cytoplasmic and nuclear RiPCA. A pre‐miR‐21 probe was used as a non‐binding control to determine background signal. (B) Viability of cells expressing SmBiT‐HT in the cytoplasm or nucleus following transfection with Lipofectamine™ RNAiMAX or TransIT‐X2®.

During our development of RiPCA, we observed that transfection with Lipofectamine™ RNAiMAX, particularly in the presence of DMSO, resulted in significant cell death (data not shown). To overcome this limitation, we turned to TransIT‐X2® (Mirus), a newly marketed transfection reagent which was demonstrated to exhibit lower cytotoxicity relative to Lipofectamine™ 2000.^[16]^ Excitingly, when used in identical quantities, transfection of RiPCA reagents with TransIT‐X2® produced equal if not greater average signal‐to‐background (S/B) in both cytoplasmic and nuclear RiPCA with pre‐let‐7d and Lin28A‐LgBiT, while requiring half the amount of Lin28‐LgBiT plasmid for transfection as compared to Lipofectamine™ RNAiMAX (Figure 2A). To verify TransIT‐X2® was less toxic in RiPCA than Lipofectamine™ RNAiMAX, the viability of cells transfected with both reagents was measured using the CellTiter‐Glo® luminescent cell viability assay. In line with data reported by Mirus, TransIT‐X2® resulted in a ∼10–18 % loss in viability in cytoplasmic and nuclear RiPCA, respectively (Figure 2B). On the other hand, transfection with Lipofectamine™ RNAiMAX resulted in ∼40–50 % loss in viability in cytoplasmic and nuclear RiPCA, respectively (Figure 2B). Subsequently, all experiments were performed with TransIT‐X2® as the transfection reagent and the assay is herein referred to as RiPCA 2.0.

### Expansion of RiPCA 2.0 for hnRNP A1 and Musashi1/2

With our optimized RiPCA 2.0 protocol in hand, we next sought to apply these conditions to determine applicability to the detection of discovered, yet poorly characterized pre‐miRNA‐protein interactions. As models, we selected hnRNP A1 and Msi1/2, which were identified via proteomics studies,^[10]^ as well as additional biological analyses,^[17]^ to bind to pre‐miRs.

Known to play a role in the regulation of alternative splicing, mRNA transport, and translation among other functions, hnRNP A1 was initially reported to bind to the terminal loop of pri‐miR‐18a, which is part of the polycistronic miRNA cluster miR‐17‐92, via CLIP analysis.^[17a]^ Recognition was found to occur via binding of hnRNP A1’s tandem RNA Recognition Motifs (RRMs) to two UAG motifs in the pri‐miR‐18a loop.^[18]^ In addition to pri‐miR‐18a, hnRNP A1 was also reported to bind to the terminal loop of pri‐let‐7s, including pri‐let‐7a‐1, via a similar UAG motif.[^17b^, ^19^] Interestingly, hnRNP A1 was found to induce opposing effects on these substrates: it enhanced pri‐miR‐18a processing by Drosha,^[17a]^ but inhibited processing of pri‐let‐7a‐1 following protein overexpression in HeLa cells.^[19]^ While proteomics experiments using pre‐miR probes confirmed these interactions, pulldown of hnRNP A1 with a pre‐miR‐18a probe was modest.^[10]^ Moreover, these experiments did not confirm an interaction with pre‐let‐7a‐1, and instead suggested that the protein may bind to other let‐7 family members, including pre‐let‐7a‐2, ‐c, ‐d, and miR‐98, in addition to other pre‐miR sequences.^[10]^ Of these, only the interaction with pre‐miR‐98 was confirmed in Jurkat cells via Western blot.^[10]^ Notably, interaction with pre‐let‐7g was also confirmed via RNA pulldown and Western blot although it was not identified via proteomics, indicating that the protein may have the capacity to bind to all let‐7s.^[10]^ Indeed, previous studies have shown the hnRNP A1 can bind to many pre‐let‐7 family members, including pre‐let‐7g, using an ELISA‐based assay employing biotinylated pre‐miR probes and HeLa cell lysate as an RBP source.^[20]^ Based on these discrepancies observed using orthogonal detection assays, hnRNP A1 became an interesting RBP for further exploration using RiPCA 2.0.

The Musashi family of proteins, Msi1 and Msi2, plays a role in maintaining stemness in undifferentiated cells and are commonly overexpressed in cancers.^[21]^ One study found that Msi1 can indirectly inhibit nuclear processing of pri‐let‐7s, most significantly pri‐miR‐98, via direct binding to Lin28 in mouse embryonic stem cells.^[17c]^ Another study identified Msi2 as an inhibitor of pri‐miR‐7 processing through formation of a complex with another RBP, Hu antigen R (HuR), in HeLa cells.^[22]^ Via proteomics, Msi1 and Msi2 were identified as binding partners of several pre‐miRNA sequences, including let‐7 family members (pre‐let‐7a‐1, ‐7a‐2, ‐7a‐3, ‐7c, ‐7f‐1, ‐7f‐2, and ‐98) and pre‐miR‐18a among others.^[10]^ Notably, interaction with pre‐miR‐7 was not observed across the 11 cell lines examined.^[10]^ As both prior reports suggested that Msi proteins act in complex with other RBPs to bind to miRNAs, along with their potential to bind and regulate let‐7s, made these proteins of interest for examination in RiPCA.

In our development of RiPCA for Lin28, we found that a pre‐let‐7d probe bearing a modified uridine at position 36 for labeling with a HT ligand produced the best signal in both Lin28A and B RiPCAs.^[13a]^ Thus, as an initial pre‐miR probe for testing hnRNP A1 and Msi1/2 in RiPCA 2.0, a pre‐let‐7d probe bearing a 5‐aminohexylacrylamino uridine modification at position 36 and conjugated to PEG4‐HT ligand (pre‐let‐7d‐36‐4Cl) was utilized (Scheme S1). As a non‐binding control probe, we used pre‐miR‐21 (pre‐miR‐21‐31‐4Cl), which we previously used for Lin28 and was deemed suitable for these RBPs as no binding has been observed or reported with hnRNP A1 or Msi1/2.[^10^, ^13a^] Of note, chemiluminescence signal generated with the pre‐miR‐21 probe was used as background, and normalized chemiluminescence signal was calculated as the ratio of signal from the target pre‐miRNA probe over that from the pre‐miR‐21 probe. We refer to this as signal‐to‐background (S/B) for the assay.

We first assessed the performance of RiPCA 2.0 with hnRNPA1‐, Msi1‐, and Msi2‐LgBiT using the same protocol previously developed for Lin28A‐LgBiT (0.5 ng/well plasmid and 33 nM RNA).^[13a]^ Unfortunately, chemiluminescence signal above background was not detected, thus requiring further optimization of transfection conditions. Increasing the amount of plasmid transfected by 4‐fold yielded modest S/B, which was used in subsequent optimization experiments (data not shown).

**Figure 3:**
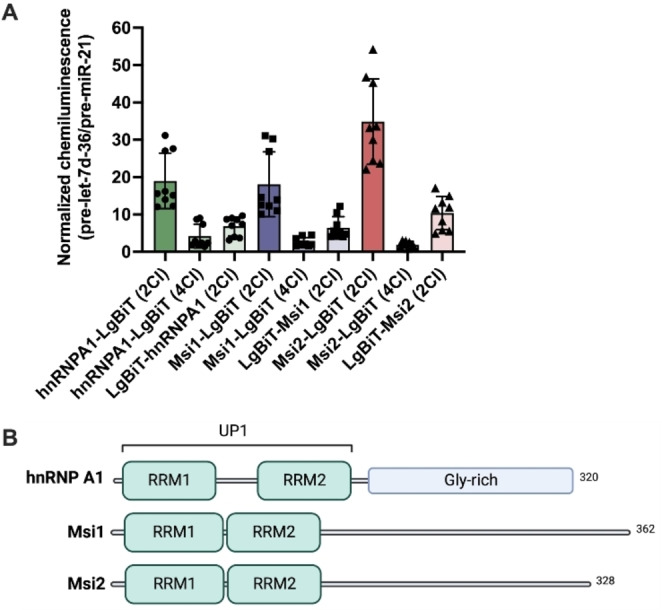
Optimization of RiPCA 2.0 for hnRNP A1, Msi1, and Msi2. (A) Identification of optimal location of the LgBiT tag and length of the linker within the HT ligand. RBP‐LgBiT represents a C‐terminally‐tagged protein. LgBiT‐RBP represents an N‐terminally‐tagged protein. 2Cl and 4Cl represent the PEG linker length on the RNA probe's HT modification. (B) Domain map of hnRNP A1, Msi1, and Msi2 proteins.

To further improve S/B, we explored the effect of PEG linker length of the HT ligand modification. RiPCA 2.0 with hnRNP A1‐, Msi1‐, and Msi2‐LgBiT was performed with previously prepared RNA probes (pre‐let‐7d and pre‐miR‐21) containing a shorter PEG2 HT ligand.^[13a]^ Importantly, decreasing the length of the PEG linker resulted in a significant increase in S/B (Figure 3A). The difference was more striking with Msi1 and Msi2 (S/B of 18.1 vs. 2.6 and 34.9 vs. 1.9, respectively) than hnRNP A1 (S/B of 19 vs. 4.2). These findings are unlike those previously observed with Lin28, where PEG length did not impact S/B.^[13a]^ Thus, this represents a point of exploration in adaptation of RiPCA to new RPI systems.

We then probed the ideal LgBiT orientation for each RBP by testing N‐ and C‐terminal constructs in RiPCA 2.0, again using pre‐miR‐21 as a non‐binding control and pre‐let‐7d as the binding sequence. While the orientation of the LgBiT tag did not significantly affect S/B generation in Lin28 RiPCA,^[13a]^ we hypothesized that there would be a preferred position for the LgBiT tag with hnRNP A1, Msi1, and Msi2 due to the organization of their RNA‐binding domains. All three proteins contain two N‐terminal RNA recognition motifs (RRMs) with a more flexible C‐terminal region (Figure 3B). Indeed, for all three RBPs, greater S/B was detected when using the construct with LgBiT located at the C‐terminus (Figure 3A). Except for LgBiT‐Msi2, the diminished S/B observed with the N‐terminally‐tagged LgBiT constructs was likely due to elevated background accompanied by a decrease in signal (Figure S3). The LgBiT‐Msi2 construct proved to be an outlier and produced greater signal than Msi2‐LgBiT, but due to excessive background, produced lower S/B (Figure S3). These results are most likely a consequence of the increased expression observed with the N‐terminally‐tagged LgBiT constructs (Figure S4), as it has been shown that higher expression of LgBiT‐tagged constructs can produce higher background signal.[^13a^, ^14^] However, considering the RRMs are located at the N‐terminus of all three proteins (Figure 3B), it is also possible that the N‐terminal LgBiT interferes with pre‐miRNA binding or that placing the LgBiT tag on the more flexible C‐terminus of these proteins allows for optimal conformation for formation of the requisite complex to enable signal production.[^18^, ^23^]

### Assessing sequence specificity of hnRNP A1, Msi1, and Msi2

We previously demonstrated that RiPCA was able to discern relative affinities of Lin28 for various pre‐miRNA sequences.^[13a]^ Having optimized RiPCA 2.0 conditions for five RBPs, we next wanted to utilize the assay to measure relative sequence specificities. Building on our previously tested library of pre‐miRNA probes, which included pre‐let‐7a‐1, ‐7d, ‐7g, and pre‐miR‐21,^[13a]^ we designed additional probes for pre‐miR‐98 and pre‐miR‐18a containing modified uridines at positions within the terminal loop for labeling with PEG2 HT ligand (Table S1), as these pre‐miRs have been identified as potential binding partners of hnRNP A1, Msi1, and Msi2.[^10^, ^17^, ^20^]

**Figure 4:**
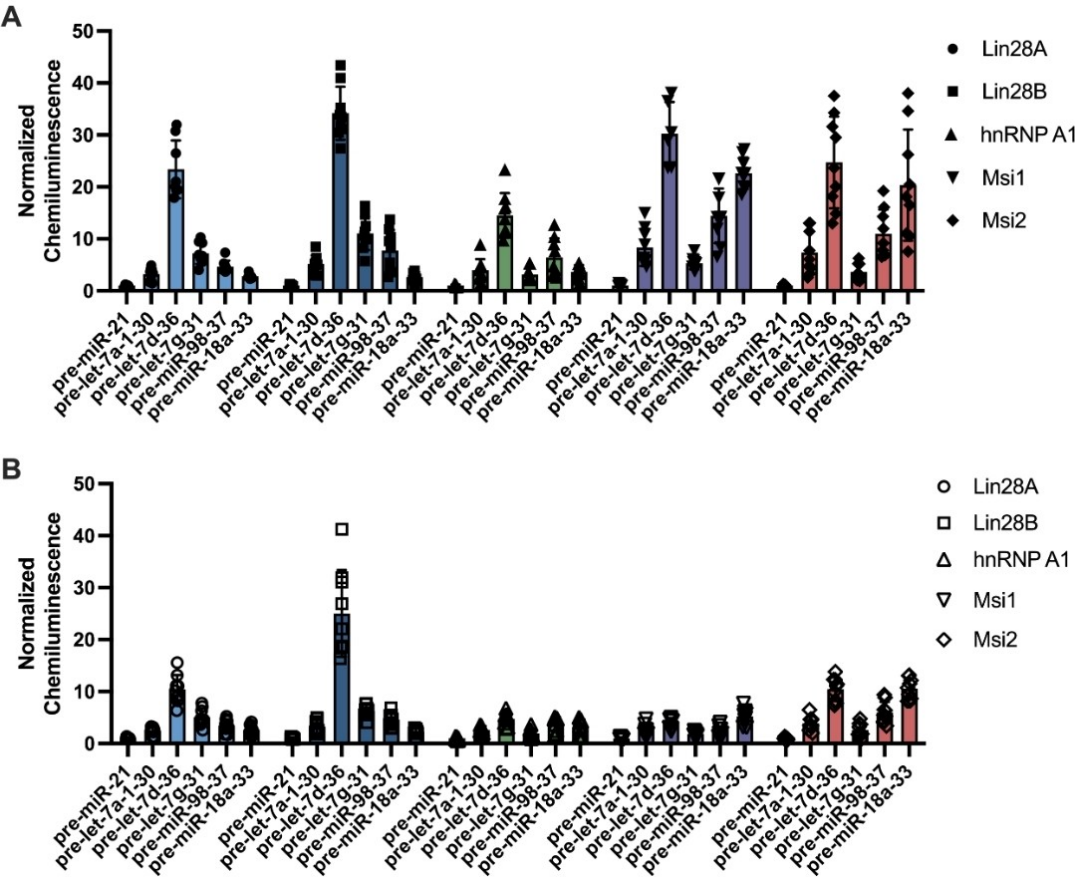
Exploring sequence specificity of C‐terminally‐tagged RBP‐LgBiT proteins using pre‐miR probes containing 2Cl HT modifications in the terminal loop. Detection in the (A) cytoplasm and (B) nucleus. The position of the uridine modification is noted as the last number in the pre‐miR probe label. Sequences can be found in Table S1.

We first confirmed that use of RiPCA 2.0 resulted in the same relative binding preferences to those previously obtained for Lin28A and Lin28B. As shown in Figure 4, our previous findings were corroborated with the greatest S/B observed with probes for pre‐let‐7d and pre‐let‐7g,^[13a]^ in‐line with established Lin28 preferences observed in CLIP‐based and RNA pulldown studies,[^11^, ^24^] as well as via proteomics.^[10]^ In both the cytoplasm and the nucleus, Lin28A and Lin28B generated modest S/B with pre‐miR‐98, but very little with pre‐miR‐18a, a non‐binding sequence,^[10]^ further confirming the ability of RiPCA to distinguish between binding and non‐binding sequences (Figure 4).

For hnRNP A1, the greatest S/B was detected in the cytoplasm with pre‐let‐7d and pre‐miR‐98 (14.5 and 6.4, respectively), whereas lower S/B was detected with reported interactors from CLIP experiments,[^17a^, ^17b^] pre‐let‐7a‐1 and pre‐miR‐18a (4.0 and 3.6, respectively) (Figure 4A). RiPCA 2.0 results do, however, agree with those obtained via proteomics where binding to pre‐let‐7d and pre‐miR‐98 was preferred over pre‐miR‐18a across the cell lines tested.^[10]^ We hypothesize that these discrepancies with CLIP results are due to either differences in experimental conditions between the methods or other cell line‐ or context‐dependent effects, which are well‐established in the field.^[11]^ In the nucleus, where hnRNP A1 is abundantly expressed and hnRNP A1‐LgBiT must outcompete endogenously expressed protein,^[25]^ the S/B detected was diminished for all sequences, with pre‐let‐7d and pre‐miR‐98 producing S/B ∼3‐ and ∼1.7‐fold lower, respectively (Figure 4B). While interactions between hnRNP A1 and select pre‐miRNA sequences were detected, these results may highlight the challenges associated with applying RiPCA to highly abundant RBPs, particularly in the nucleus.

**Figure 5:**
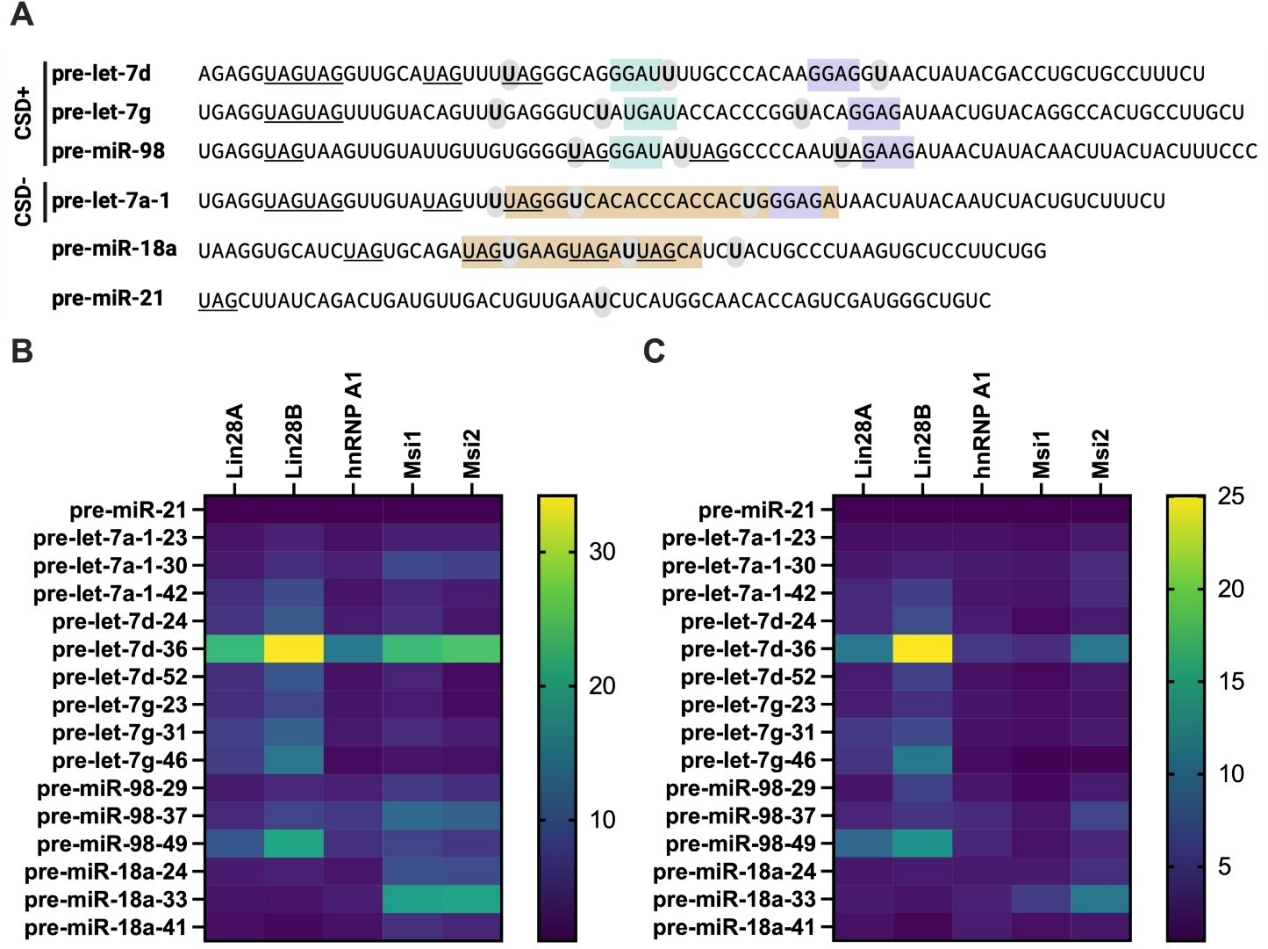
Exploring detection of site‐specific binding in RiPCA 2.0. (A) Pre‐miRNA sequences used to generate RiPCA probes. The Lin28 CSD and ZKD binding sites are highlighted in light green and purple, respectively. The hnRNP A1 footprint is highlighted in light tan. UAG motifs are underlined. The sites of the modified uridines are highlighted in grey and bolded. Heat map of Lin28A‐, Lin28B‐, hnRNP A1‐, Msi1‐, and Msi2‐LgBiT binding to the library of pre‐miRNA probes in the (B) cytoplasm and (C) nucleus. Legend indicates increasing S/B, as defined by signal divided by the average of pre‐miR‐21 signal, from blue to yellow. Position of the uridine modification is noted as the last number in the probe label. Normalized chemiluminescence data for each RBP can be found in Figure S5.

For Msi1 and Msi2, RiPCA 2.0 results were again aligned with those from proteomics.^[10]^ In the cytoplasm, we observed strong signal produced with both pre‐miR‐18a and pre‐miR‐98 (Figure 4A). With respect to pre‐let‐7s, overall, the highest chemiluminescence signal was observed with pre‐let‐7d, while modest signal was detected with pre‐let‐7a‐1 and ‐7g (Figure 4B). Aside from pre‐let‐7a‐1, optimal S/B was observed with those sequences containing the greatest number of UAG sites (Figure 5A). In the nucleus, Msi2 retained similar relative binding preferences, whereas Msi1 S/B was an average of ∼4.9‐fold lower than in the cytoplasm (Figure 4B). We hypothesize that this is due to reduced expression of Msi1‐LgBiT in the nucleus, as Msi1 is primarily localized in the cytoplasm.^[26]^ Overall, these data provide evidence for the application of RiPCA 2.0 to investigate the binding profiles of RBPs.

### Exploring molecular interactions in RiPCA 2.0

For our initial development of RiPCA,^[13a]^ as well as for our extended probe library described (*vida supra*), we focused on a single site of uridine modification, which may not be optimal for our RBPs‐of‐interest. Thus, we were curious to investigate the influence of the location of the modified uridine, and thus the site of HT ligand conjugation relative to the RBP recognition sites within the pre‐miRNA probe, on detection using RiPCA 2.0. To do so, we designed additional probes for each of our RBP‐LgBiT proteins (Table S1 and Figure 5A).

The individual RNA‐binding domains of Lin28 are a cold shock domain (CSD) and tandem zinc knuckle domains (ZKD), which bind GNGAY and GGAG motifs, respectively (Figure 5A).[^7a^, ^24^] In the original library of pre‐let‐7 probes, the modified uridine residue was placed no further than 2 nucleotides from the CSD binding site, which is known to exhibit higher binding affinity for pre‐let‐7s.[^7a^, ^24^, ^27^] To expand this library, a probe with the modified uridine close to the ZKD binding site was also designed (Figure 5A).

The consensus binding sequence of hnRNP A1 was previously identified as UAGGGA/U using selective amplification experiments (SELEX).^[28]^ High‐throughput sequencing analysis of equilibrium binding (HTS‐EQ) confirmed the YAG consensus motif, yet also revealed that hnRNP A1 can bind to an array of sequences dependent upon the nucleotides surrounding the YAG motif.^[29]^ In footprinting experiments, hnRNP A1 was found to interact within the terminal loop of reported binders, pre‐let‐7a‐1 and pre‐miR‐18a, which include UAG motifs (Figure 5A).^[17b]^ The Msi proteins have similarly been shown to bind to an r(UAG) motif, which is contained within the hnRNP A1 consensus sequence, potentially providing an explanation for the overlap in pre‐miRNA binding.^[23]^ We, therefore, designed probes to test binding to various UAG motifs throughout the terminal loops encompassing both the putative hnRNP A1 and Msi1/2 binding sites (Figure 5A). Prior to analysis, we first confirmed that all probes could be labeled to a similar extent by SmBiT‐HT by performing electrophoretic mobility shift assays (EMSA) with pre‐miRNA probes and purified SmBiT‐HT protein (Figure S6).

With our library of pre‐miRNA probes in hand, we performed RiPCA 2.0 in the cytoplasm and nucleus with each RBP (Figures 5B, 5C, and S5). For most RBP/probe combinations, the greatest S/B was produced with probes containing the uridine modification towards the middle of the terminal loop (Figures 5 and S5). Notably, for the Lin28 proteins, pre‐let‐7a‐1, ‐7g‐ and ‐98 were exceptions. For these pre‐let‐7s, unlike pre‐let‐7d, the highest S/B was observed using probes in which the HT ligand‐labeled residue was just upstream of the ZKD binding site (Figures 5 and S5). This variance highlights potential differences between the loop structures of these pre‐let‐7s, as well as the sensitivity of RiPCA to the location of modification site on the RNA probe for subsequent RBP binding and complementation of SmBiT and LgBiT to reconstitute NanoLuc for detection of the RPI.

Compared to the other four RBPs tested, hnRNP A1 produced the lowest S/B and imperceptible site specificity in‐line with its reported promiscuity^[29]^ (Figures 5 and S5). While low S/B across the tested probes may be due to overwhelming competition with endogenous hnRNP A1,^[25]^ it could also be due to short residence time of the protein on the RNAs or lower affinity for these hairpins in comparison to its preferred RNA substrates.[^17a^, ^29^] While Msi1 and Msi2 showed strong sequence specificity, no site selectivity was detected, as the greatest S/B was detected with the uridine modification in the middle of each terminal loop regardless of proximity to a UAG motif (Figures 5 and S5). As structural information regarding the interactions of Msi1/2 with pre‐miRNA substrates is not available, we cannot rule out that this site of modification may enable binding of the Msi1/2 tandem RRMs (Figure 3B). Overall, given the general success of RNA probes containing a mid‐loop modification, we hypothesize that placing the uridine modification in the middle of the terminal loop may provide optimal flexibility allowing for enhanced complementation of SmBiT and LgBiT following binding of the RNA and RBP and should be taken into consideration when designing additional probes to detect RPIs with stem‐loop RNA structures.

## Conclusion

The development of tools for the study of cellular RPIs promises to advance our ability to validate and characterize, as well as work towards discovering modulators of these interactions. The assay reported in the present work contributes to this effort as it was demonstrated to enable detection of pre‐miRNA binding by hnRNP A1, Msi1, and Msi2, and determine the sequence specificity of these RBPs. Most importantly, through these efforts, we reveal new insights into design considerations for the development of future RiPCA 2.0 RNA probes and assays for investigating additional RPI systems. Beyond being integral to miRNA biology, hairpin loop structures are found ubiquitously throughout the transcriptome and are important for RNA function.^[30]^ The broad adaptability of RiPCA 2.0 for the detection of a variety of pre‐miRNA‐protein interactions provides evidence of the utility of this technology in studying additional motif‐RBP interactions. Future efforts in exploring RPIs involving diverse classes of RNAs, including mRNAs, lncRNAs, and expanded repeats, will enable us to probe this further.

Previous efforts to utilize the original RiPCA protocol to detect pre‐let‐7d/Lin28 inhibition were hindered by the combined toxicity of transfection with Lipofectamine™ RNAiMAX and treatment of a compound dissolved in DMSO (Figure S7). Therefore, the optimization of the protocol to minimize cell death should allow use of RiPCA 2.0 as a platform for detecting RPI modulation in live cells by small molecules. These efforts are on‐going in the lab and will be reported in due course.

## Experimental Section

**Synthesis of pre‐miRNA Probes for RiPCA**: Pre‐miRNA probes bearing a 5‐aminohexylacrylamino uridine modification and biotin appended to the 5’ end by an 18‐atom spacer (1.0 mM in 100 mM phosphate buffer, pH 8.0) were treated with an equivalent volume of HaloTag ligand (10 mM in DMSO of PEG2‐ or PEG4‐ligand). Reactions proceeded at 25 °C for 1 h. Labelled RNAs were then precipitated by the addition of 0.11× volume of 3.0 M sodium acetate (pH 5.2) and 4 volume equivalents of cold ethanol, and pelleted at 20,000×g for 40 min at 4 °C. Pellets were subsequently re‐suspended in 100 mM phosphate buffer (pH 8.0) at a concentration of 1.0 mM and stored at −80 °C.

**RiPCA 2.0 protocol**: Flp‐In‐293 cells stably expressing a SmBiT‐HT protein were reverse transfected using TransIT‐X2® Reagent. Cells were passaged approximately 10 times, and no more than 15 times, before returning to low passage stocks. Solution B for each condition was prepared by adding in order DNA (volumes provided in Table S1), 0.45 μL of 25 μM RNA probe, and 1.126 μL TransIT‐X2® to 37.5 μL room temperature Opti‐MEM™. Solution B was mixed by being briefly vortexed and was briefly centrifuged prior to ∼15 min incubation at room temperature while cells were harvested. Cells were harvested as and counted as described above. Harvested cells were used to prepare Solution C (300 μL×(n+1) of 100,000 cells/mL) and 300 μL of Solution C was added to Solution B. Solution B+C was mixed by pipetting up and down before plating 100 μL per well, 3 wells per condition, in a white‐bottom, tissue culture‐treated 96‐well plate (Corning cat #3917). The plate was incubated in a humidified incubator (37 °C and 5 % CO_2_) for 24 h. After incubation, the media was removed and replaced with 100 μL room temperature Opti‐MEM™ and treated with 25 μL NanoGlo Live Cell Reagent diluted 1 : 20 according to the manufacturer's recommendation. All chemiluminescence data was collected immediately on a BioTek Cytation3 plate reader.

Please see Supporting Information (Figure S2) for additional details.

## Conflict of interest

The authors declare no conflict of interest.

1

## Supporting information

As a service to our authors and readers, this journal provides supporting information supplied by the authors. Such materials are peer reviewed and may be re‐organized for online delivery, but are not copy‐edited or typeset. Technical support issues arising from supporting information (other than missing files) should be addressed to the authors.

Supporting Information

## Data Availability

The data that support the findings of this study are available from the corresponding author upon reasonable request.
