# Supplementary material for: Optimization of RiPCA for the Live‐Cell Detection of Pre‐MicroRNA‐Protein Interactions
Source: Chembiochem. 2022 Nov 22;23(24):e202200508. doi: 10.1002/cbic.202200508 (PMC9831681; doi:10.1002/cbic.202200508)
Supplement: Supplementary file 1 — Supporting Information [file CBIC-23-0-s001.pdf]

ChemBioChem

Supporting Information

Optimization of RiPCA for the Live-Cell Detection of Pre-MicroRNA-Protein Interactions

Sydney L. Rosenblum and Amanda L. Garner*

**A. General Materials and Methods**	Page S2
**B. Cloning**	Pages S2−S5
**C. RiPCA Protocols**	Pages S5−S8
**D. CellTiter-Glo® Assay**	Page S8
**E. EMSA**	Page S8
**F. Supplemental Figures**	Pages S9−S12
**G. References**	Page S12

A. General Materials and Methods

Materials:

Chemically synthesized pre-microRNAs (deprotected, desalted and HPLC purified) containing 5-aminohexylacrylamino uridine (5-LC-N-U) modifications and biotin attached to the 5'-end of the sequence by an 18-atom spacer were purchased from Horizon Discovery Biosciences (formerly Dharmacon) and used as received for the labeling reaction. HaloTag Succinimidyl Ester (O2) and (O4) Ligands were purchased from Promega and used as received (cat #1691 and #P6751). Note that the HaloTag Succinimidyl Ester Ligands should be dissolved and immediately portioned into single-use aliquots stored at -80 °C to avoid degradation. The generation of Flp-In™-293 cells stably expressing SmBiT-HT or SmBiT-HT-cNLS was previously reported.[1] The Nano-Glo Live Cell Assay System was purchased from Promega and used as received (cat #N2012). Transfection reagents Lipofectamine™ RNAiMAX (Invitrogen cat #13778100) and TransIT-X2® (Mirus cat #6000) were used as received. Cell Titer Glo was purchased from Promega and used as received (cat #G7570).

Cell Culture:

Flp-In™-293 cells stably expressing either SmBiT-HaloTag or SmBiT-HaloTag-NLS were cultured in DMEM (Corning cat #10-017-CV) supplemented with 10% FBS (Atlanta Biologicals S11550), L-glutamine (Gibco cat #25030081), and hygromycin B (100 µg/mL) (Gibco cat #10687010) at 37 °C with 5% CO_2 in a humidified incubator, passaged at least once before use for an experiment. HEK 293 cells were maintained in DMEM (Corning cat #10-017-CV) supplemented with 10% FBS (Atlanta Biologicals S11550), and L-glutamine (Gibco cat #25030081) at 37 °C with 5% CO_2 in a humidified incubator, passaged at least once before use for an experiment. Cells were passaged using Trypsin-EDTA (0.25%) (Gibco cat #25300054) approximately 10 times, and no more than 15 times, before returning to low passage stocks. To count cells, cells were harvested and 10 µL of the cell suspension was mixed with 10 µL Trypan Blue (Gibco cat #15250061) ([final] = 0.2% trypan blue) and counted using a hemocytometer.

General assay and data analysis methods:

Chemiluminescence data was collected on a BioTek Cytation3 plate reader. All data was analyzed using GraphPad Prism version 9.0.0 for Mac OS X (GraphPad Software, www.graphpad.com). All normalized chemiluminescence is reported as the signal of each well divided by the average signal of triplicate pre-miR-21 wells.

B. Cloning

hnRNPA1-LgBiT cloning. A synthetic human hnRNPA1 gene fragment was purchased from Twist Bioscience and inserted at the C-terminal position[1b] using standard cloning techniques with KpnI and AsiSI restriction enzymes. Gene fragment contains a Kozak sequence at the N-terminus.

Gene Fragment:
5'GGTACCGCCACCATGTCTAAGTCAGAGTCTCCTAAAGAGCCCGAACAGCTGAGGAAGCT
CTTCATTGGAGGGTTGAGCTTTGAAACAACTGATGAGAGCCTGAGGAGCCATTTTGAGCA
ATGGGGAACGCTCACGGACTGTGTGGTAATGAGAGATCCAAACACCAAGCGCTCCAGGG
GCTTTGGGTTTGTCACATATGCCACTGTGGAGGAGGTGGATGCAGCTATGAATGCAAGGC
CACACAAGGTGGATGGAAGAGTTGTGGAACCAAAGAGAGCTGTCTCCAGAGAAGATTCT
CAAAGACCAGGTGCCCACTTAACTGTGAAAAAGATATTTGTTGGTGGCATTAAAGAAGAC

ACTGAAGAACATCACCTAAGAGATTATTTGAACAGTATGGAAAAATTGAAGTGATTGAA
ATCATGACTGACCGAGGCAGTGGCAAGAAAAGGGGCTTTGCCTTTGTAACCTTTGACGAC
CATGACTCCGTGGATAAGATTGTCATTCAGAAATACCATACTGTGAATGGCCACAACTGT
GAAGTTAGAAAAGCCCTGTCAAAGCAAGAGATGGCTAGTGCTTCATCCAGCCAAAGAGG
TCGAAGTGGTTCTGGAAACTTTGGTGGTGGTCGTGGAGGTGGTTTCGGTGGGAATGACAA
CTTCGGTCGTGGAGGAAACTTCAGTGGTCGTGGTGGCTTTGGTGGCAGCCGTGGTGGTGG
TGGATATGGTGGCAGTGGGGATGGCTATAATGGATTTGGTAATGATGGAAGCAATTTTGG
AGGTGGTGGAAGCTACAATGATTTTGGGAATTACAACAATCAGTCTTCAAATTTTGGACC
CATGAAGGGAGGAAATTTTGGAGGCAGAAGCTCTGGCCCCTATGGCGGTGGAGGCCAAT
ACTTTGCAAAACCACGAAACCAAGGTGGCTATGGCGGTTCCAGCAGCAGCAGTAGCTATG
GCAGTGGCAGAAGATTTGCGATCGC

LgBiT-hnRNPA1 cloning. A synthetic human hnRNPA1 gene fragment was purchased from Twist Bioscience and inserted at the N-terminal position[1b] using standard cloning techniques with XhoI and XbaI restriction enzymes.

Gene Fragment:
5'CTCGAGATGTCTAAGTCAGAGTCTCCTAAAGAGCCCGAACAGCTGAGGAAGCTCTTCAT
TGGAGGGTTGAGCTTTGAAACAACTGATGAGAGCCTGAGGAGCCATTTTGAGCAATGGGG
AACGCTCACGGACTGTGTGGTAATGAGAGATCCAAACACCAAGCGCTCCAGGGGCTTTGG
GTTTGTCACATATGCCACTGTGGAGGAGGTGGATGCAGCTATGAATGCAAGGCCACACAA
GGTGGATGGAAGAGTTGTGGAACCAAAGAGAGCTGTCTCCAGAGAAGATTCTCAAAGAC
CAGGTGCCCACTTAACTGTGAAAAAGATATTTGTTGGTGGCATTAAAGAAGACACTGAAG
AACATCACCTAAGAGATTATTTGAACAGTATGGAAAAATTGAAGTGATTGAAATCATGA
CTGACCGAGGCAGTGGCAAGAAAAGGGGCTTTGCCTTTGTAACCTTTGACGACCATGACT
CCGTGGATAAGATTGTCATTCAGAAATACCATACTGTGAATGGCCACAACTGTGAAGTTA
GAAAAGCCCTGTCAAAGCAAGAGATGGCTAGTGCTTCATCCAGCCAAAGAGGTCGAAGT
GGTTCTGGAAACTTTGGTGGTGGTCGTGGAGGTGGTTTCGGTGGGAATGACAACTTCGGT
CGTGGAGGAAACTTCAGTGGTCGTGGTGGCTTTGGTGGCAGCCGTGGTGGTGGTGGATAT
GGTGGCAGTGGGGATGGCTATAATGGATTTGGTAATGATGGAAGCAATTTTGGAGGTGGT
GGAAGCTACAATGATTTTGGGAATTACAACAATCAGTCTTCAAATTTTGGACCCATGAAG
GGAGGAAATTTTGGAGGCAGAAGCTCTGGCCCCTATGGCGGTGGAGGCCAATACTTTGCA
AAACCACGAAACCAAGGTGGCTATGGCGGTTCCAGCAGCAGCAGTAGCTATGGCAGTGG
CAGAAGATTTTAATCTAGA

Msi1-LgBiT cloning. A synthetic human Musashi 1 (Msi1) gene fragment was purchased from Twist Bioscience and inserted at the C-terminal position[1b] using standard cloning techniques with HindIII and AsiSI restriction enzymes. Gene fragment contains a Kozak sequence at the N-terminus.

Gene Fragment:
5'AAGCTTGCCACCATGGAGACTGACGCGCCCCAGCCCGGCCTCGCCTCCCCGGACTCGCC
GCACGACCCCTGCAAGATGTTCATCGGGGGACTCAGTTGGCAGACTACGCAGGAAGGGCT
GCGCGAATACTTCGGCCAGTTCGGGGAGGTGAAGGAGTGTCTGGTGATGCGGGACCCCCT
GACCAAGAGATCCAGGGGTTTCGGCTTCGTCACTTTCATGGACCAGGCGGGGGTGGATAA
AGTGCTGGCGCAATCGCGGCACGAGCTCGACTCCAAAACAATTGACCCTAAGGTGGCCTT
CCCTCGGCGAGCACAGCCCAAGATGGTGACTCGAACGAAGAAGATCTTTGTGGGGGGGCT
GTCGGTGAACACCACGGTGGAGGACGTGAAGCAATATTTTGAGCAGTTTGGGAAGGTGG
ACGACGCCATGCTGATGTTTGACAAAACCACCAACCGGCACCGAGGGTTCGGGTTTGTCA

CGTTTGAGAGTGAGGACATCGTGGAGAAAGTGTGTGAAATTCATTTTCATGAAATCAACA
ACAAAATGGTGGAATGTAAGAAAGCTCAGCCAAAGGAGGTGATGTCGCCAACGGCTCA
GCCCGGGGGAGGTCTCGAGTCATGCCCTACGGAATGGACGCCTTCATGCTGGGCATCGGC
ATGCTGGGTTACCCAGGTTTCCAAGCCACAACCTACGCCAGCCGGAGTTATACAGGCCTC
GCCCCTGGCTACACCTACCAGTTCCCCGAATTCCGTGTAGAGCGGACCCCTCTCCCGAGCG
CCCCAGTCCTCCCCGAGCTTACAGCCATTCCTCTCACTGCCTACGGACCAATGGCGGCGGC
AGCGGCGGCAGCGGCTGTGGTTCGAGGGACAGGCTCTCACCCCTGGACGATGGCTCCCCC
TCCAGGTTCGACTCCCAGCCGCACAGGGGGCTTCCTGGGGACCACCAGCCCCGGCCCCAT
GGCCGAGCTCTACGGGGCGGCCAACCAGGACTCGGGGGTCAGCAGTTACATCAGCGCCG
CCAGCCCTGCCCCCAGCACCGGCTTCGGCCACAGTCTTGGGGGCCCTTTGATTGCCACAGC
CTTCACCAATGGGTACCACGCGATCGC

LgBiT-Msi1 cloning. A synthetic human Msi1 gene fragment was purchased from Twist Bioscience and inserted at the N-terminal position[1b] using standard cloning techniques with EcoRV and XbaI restriction enzymes.

Gene Fragment:
5’GATATCTTATGGAGACTGACGCGCCCCAGCCCGGCCTCGCCTCCCCGGACTCGCCGCAC
GACCCCTGCAAGATGTTCATCGGGGGACTCAGTTGGCAGACTACGCAGGAAGGGCTGCGC
GAATACTTCGGCCAGTTCGGGGAGGTGAAGGAGTGTCTGGTGATGCGGGACCCCCTGACC
AAGAGATCCAGGGGTTTCGGCTTCGTCACTTTCATGGACCAGGCGGGGGTGGATAAAGTG
CTGGCGCAATCGCGGCACGAGCTCGACTCCAAAACAATTGACCCTAAGGTGGCCTTCCCT
CGGCGAGCACAGCCCAAGATGGTGACTCGAACGAAGAAGATCTTTGTGGGGGGGCTGTC
GGTGAACACCACGGTGGAGGACGTGAAGCAATATTTTGAGCAGTTTGGGAAGGTGGACG
ACGCCATGCTGATGTTTGACAAAACCACCAACCGGCACCGAGGGTTCGGGTTTGTCACGT
TTGAGAGTGAGGACATCGTGGAGAAAGTGTGTGAAATTCATTTTCATGAAATCAACAACA
AAATGGTGGAATGTAAGAAAGCTCAGCCAAAGGAGGTGATGTCGCCAACGGGCTCAGCC
CGGGGGAGGTCTCGAGTCATGCCCTACGGAATGGACGCCTTCATGCTGGGCATCGGCATG
CTGGGTTACCCAGGTTTCCAAGCCACAACCTACGCCAGCCGGAGTTATACAGGCCTCGCC
CCTGGCTACACCTACCAGTTCCCCGAATTCCGTGTAGAGCGGACCCCTCTCCCGAGCGCCC
CAGTCCTCCCCGAGCTTACAGCCATTCCTCTCACTGCCTACGGACCAATGGCGGCGGCAG
CGGCGGCAGCGGCTGTGGTTCGAGGGACAGGCTCTCACCCCTGGACGATGGCTCCCCCTC
CAGGTTCGACTCCCAGCCGCACAGGGGGCTTCCTGGGGACCACCAGCCCCGGCCCCATGG
CCGAGCTCTACGGGGCGGCCAACCAGGACTCGGGGGTCAGCAGTTACATCAGCGCCGCCA
GCCCTGCCCCCAGCACCGGCTTCGGCCACAGTCTTGGGGGCCCTTTGATTGCCACAGCCTT
CACCAATGGGTACCACTGATCTAGA

Msi2-LgBiT cloning. Musashi 2 (Msi2) was amplified from a purchased plasmid, Msi2 variant 1 in pFN21A (Promega), and inserted into pcDNA3 containing LgBiT inserted at the C-terminal position[1b] using standard cloning techniques with KpnI and AsiSI restriction enzymes. Primers insert a Kozak sequence on the N-terminus.

Primers:

5’ GTACGGTACCGCCACCATGGAGGCAAATGGGAGCCAAG
5’ GTCACGGCGATCGCATGGTATCCATTTGTAAAGGCC

LgBiT-Msi2 cloning. Human Msi 2 was amplified from pcDNA3 + Msi2-Lg and ligated into a pcDNA3 construct with LgBiT inserted at the N-terminal position using standard cloning techniques with XhoI and EcoRI restriction enzymes.

Primers:

5’ TCTCCTCGAGATGGAGGCAAATGGGAGCC
5’ CAGTGAATTCTCAATGGTATCCATTTGTAAAGGCCG

C. RiPCA Protocols

Bioconjugation:

A pre-miRNA probe bearing a 5-aminohexylacrylamino uridine modification (5-LC-N-U) and biotin appended to the 5’ end by an 18-atom spacer (Scheme S1) (1.0 mM in 100 mM phosphate buffer, pH 8.0) was mixed with an equivalent volume of HaloTag ligand (10 mM in DMSO of PEG2- or PEG4-ligand). The reaction was then allowed to proceed at 25 °C for 1 h. pre-miRNA-Cl was precipitated by the addition of 0.11× volume of 3.0 M sodium acetate (pH 5.2) and 4 volume equivalents of cold ethanol, and pelleted at 20,000 × g for 40 min at 4 °C. The pellet was then re-suspended in 100 mM phosphate buffer (pH 8.0) at a concentration of 1.0 mM and stored at -80 °C.

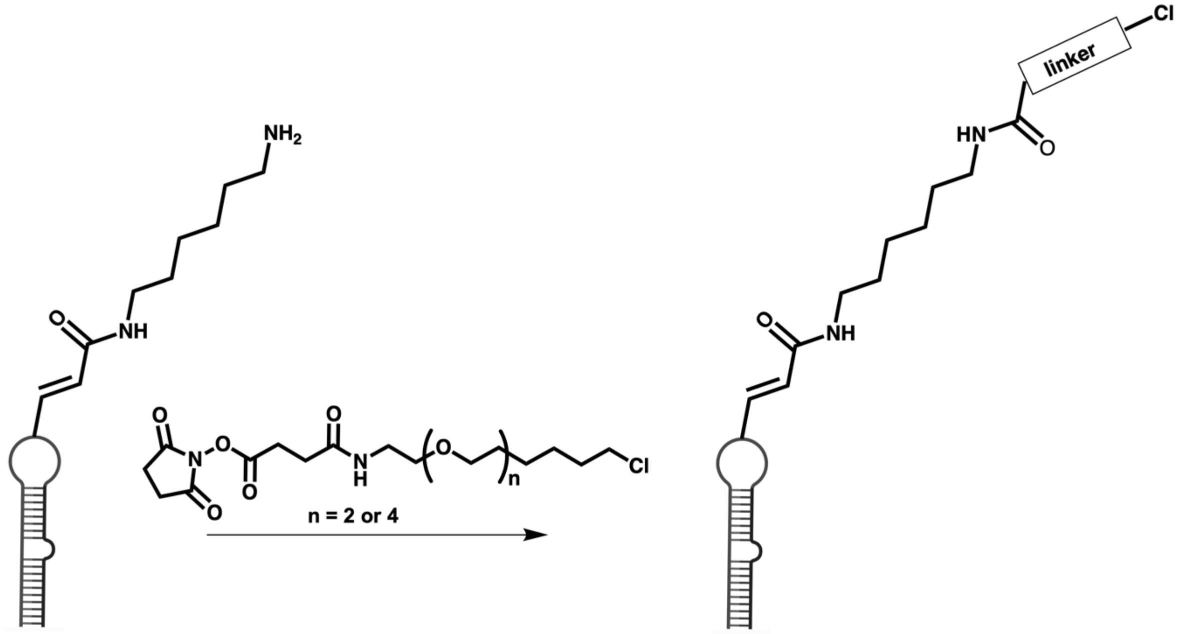

Scheme S1. Labeling of RNAs containing a 5-aminohexylacrylamino uridine modification with HaloTag ligands via NHS ester coupling. RNA sequences used, including sites of uridine modification, can be found in Table S1.

Table S1. Sequences of pre-miR probes used in RiPCA

pre-miR Probe	Sequence
pre-miR-21-31	UAGCUUAUCAGACUGAUGUUGACUGUUGAA**(5-LC-N-U)**CUCAUGGCAACACCAGUCGAUGGGCUGUC
pre-let-7a-1-23	UGAGGUAGUAGGUUGUAUAGUU(**5-LC-N-U**)UAGGGUCACACCCACCACUGGGAGAUAACUAUACAAUCUACUGUCUUUCU
pre-let-7a-1-30	UGAGGUAGUAGGUUGUAUAGUUUUAGGG(**5-LC-N-U**)CACACCCACCACUGGGAGAUAACUAUACAAUCUACUGUCUUUCU
pre-let-7a-1-42	UGAGGUAGUAGGUUGUAUAGUUUUAGGGUCACACCCACCAC(**5-LC-N-U**)GGGAGAUAACUAUACAAUCUACUGUCUUUCU
pre-let-7d-24	AGAGGUAGUAGGUUGCAUAGUUU(**5-LC-N-U**)AGGGCAGGAUUUUGCCCACAAGGAGGUAACUAUACGACCUGCUGCCUUUCU

pre-let-7d-36	AGAGGUAGUAGGUUGCAUAGUUUUAGGAGCAGGGAU(**5-LC-N-U**)UUGCCC ACAAGGAGGUAACUAUACGACCUGCUGCCUUUCU
pre-let-7d-52	AGAGGUAGUAGGUUGCAUAGUUUUAGGAGCAGGGAUUUUGCCCACAAGG AGG(**5-LC-N-U**)AACUAUACGACCUGCUGCCUUUCU
pre-let-7g-23	UGAGGUAGUAGUUUGUACAGUU(**5-LC-N-U**)GAGGGUCUAUGAUACCACCC GGUACAGGAGAUAACUGUACAGGCCACUGCCUUGCU
pre-let-7g-31	UGAGGUAGUAGUUUGUACAGUUUGAGGGUC(**5-LC-N-U**)AUGAUACCACCC GGUACAGGAGAUAACUGUACAGGCCACUGCCUUGCU
pre-let-7g-46	UGAGGUAGUAGUUUGUACAGUUUGAGGGUCUAUGAUACCACCCGG(**5-LC-N-U**)ACAGGAGAUAACUGUACAGGCCACUGCCUUGCU
pre-miR-98-29	UGAGGUAGUAAGUUGUAUUGUUGUGGGG(**5-LC-N-U**)AGGGAUAUUAGGCC CCAAUUAGAAGAUAACUAUACAACUUACUACUUUCCC
pre-miR-98-37	UGAGGUAGUAAGUUGUAUUGUUGUGGGGUAGGGA(**5-LC-N-U**)AUUAGGCC CCAAUUAGAAGAUAACUAUACAACUUACUACUUUCCC
pre-miR-98-49	UGAGGUAGUAAGUUGUAUUGUUGUGGGGUAGGGAUAUUAGGCCCCAAU(**5-LC-N-U**)AGAAGAUAACUAUACAACUUACUACUUUCCC
pre-miR-18a-24	UAAGGUGCAUCUAGUGCAGAUAG(**5-LC-N-U**)GAAGUAGAUUAGCAUCUAC UGCCCUAAGUGCUCCUUCUGG
pre-miR-18a-33	UAAGGUGCAUCUAGUGCAGAUAGUGAAGUAGA(**5-LC-N-U**)UAGCAUCUAC UGCCCUAAGUGCUCCUUCUGG
pre-miR-18a-41	UAAGGUGCAUCUAGUGCAGAUAGUGAAGUAGAUUAGCAUC(**5-LC-N-U**)AC UGCCCUAAGUGCUCCUUCUGG

Lipofectamine™ RNAiMAX protocol:
Flp-In-293 cells stably expressing a SmBiT-HT protein were reverse transfected using Lipofectamine™ RNAiMAX Transfection Reagent. Cells were passaged approximately 10 times, and no more than 15 times, before returning to low passage stocks. To test "n" number of conditions, Solution A was prepared by combining 50 × (n+1) µL of room temperature Opti-MEM™ and 2.4 × (n+1) µL plasmid encoding selected RBP-LgBiT fusion. Solution B was prepared by adding pre-miRNA-Cl and plasmid (final concentrations 0.3 µM and 0.195 ng/µL, respectively) to 50 µL Opti-MEM™ for each separate condition to be tested. Solution B was mixed with 50 µL of Solution A to yield Solution A+B, which was incubated for at least 15 min at room temperature while cells were harvested. Cells were harvested as and counted as described above. Harvested cells were used to prepare Solution C, which was composed of 400 µL of 100,000 cells/mL. Solution C was mixed with 50 µL of Solution A+B and plated 100 µL per well, four wells per condition, in a white-bottom, tissue culture-treated 96-well plate (Corning cat #3917). The plate was incubated in a humidified incubator (37 °C and 5% CO_2) for 24 h. After incubation, the media was removed and replaced with 100 µL room temperature Opti-MEM™ and treated with 25 µL NanoGlo Live Cell Reagent diluted 1:20 according to the manufacturer's recommendation. All chemiluminescence data was collected immediately on a BioTek Cytation3 plate reader. Additional information our published protocol.[1a]

Representative calculations based on an assay for n = 5 conditions:
<u>Solution A: Prepared for n+1= 6</u>
6 x 50 µL → 300 µL Opti-MEM™
6 x 2.4 µL → 14.4 µL Lipofectamine™ RNAiMAX
<u>Solution B:</u>
50 µL Opti-MEM™
2.5 µL 3.9 ng/µL RBP-LgBiT plasmid

0.3 µL 50 µM pre-miRNA-Cl

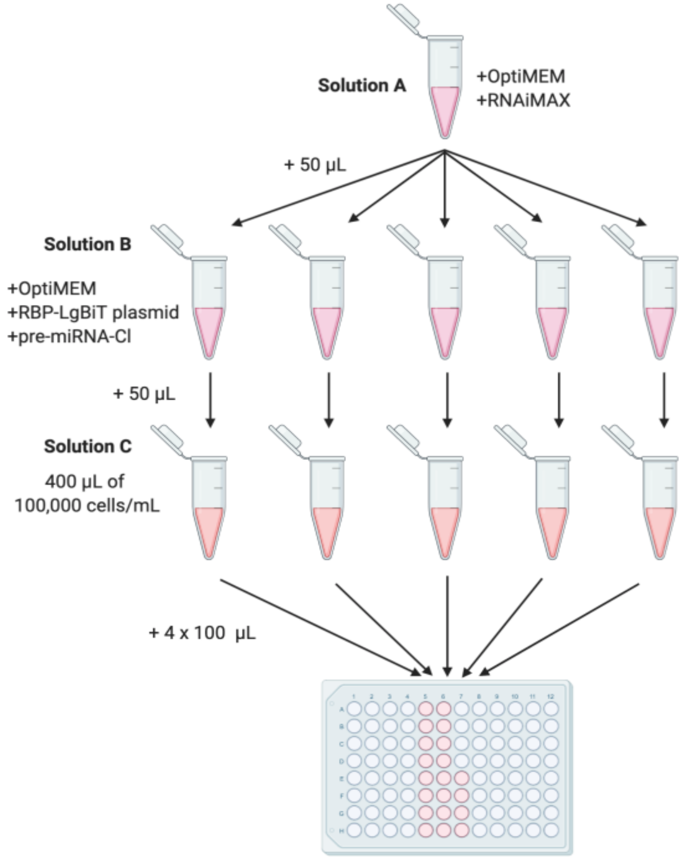

Figure S1. Lipofectamine™ RNAiMAX transfection workflow.

TransIT-X2® Protocol:
Flp-In-293 cells stably expressing a SmBiT-HT protein were reverse transfected using TransIT-X2® Reagent. Cells were passaged approximately 10 times, and no more than 15 times, before returning to low passage stocks. Solution B for each condition was prepared by adding in order DNA (volumes provided in Table S1), 0.45 µL of 25 µM RNA probe, and 1.126 µL TransIT-X2® to 37.5 µL room temperature Opti-MEM™. Solution B was mixed by briefly vortexing and was briefly centrifuged prior to ~15 min incubation at room temperature while cells were harvested. Cells were harvested as and counted as described above. Harvested cells were used to prepare Solution C (300 µL × (n+1) of 100,000 cells/mL) and 300 µL of Solution C was added to Solution B. Solution B+C was mixed by pipetting up and down before plating 100 µL per well, 3 wells per condition, in a white-bottom, tissue culture-treated 96-well plate (Corning cat #3917). The plate was incubated in a humidified incubator (37 °C and 5% CO_2) for 24 h. After incubation, the media was removed and replaced with 100 µL room temperature Opti-MEM™ and treated with 25 µL NanoGlo Live Cell Reagent diluted 1:20 according to the manufacturer's recommendation. All chemiluminescence data was collected immediately on a BioTek Cytation3 plate reader.

RiPCA 2.0 conditions based on RBP plasmid:

	Volume of DNA	Concentration of DNA	Amount of DNA per well
**Lin28A/B**	0.47 µL	3.9 ng/µL	~0.5 ng
**hnRNP A1**	1.876 µL	3.9 ng/µL	~2 ng
**Msi1/2**	1.726 µL	3.9 ng/µL	~2 ng

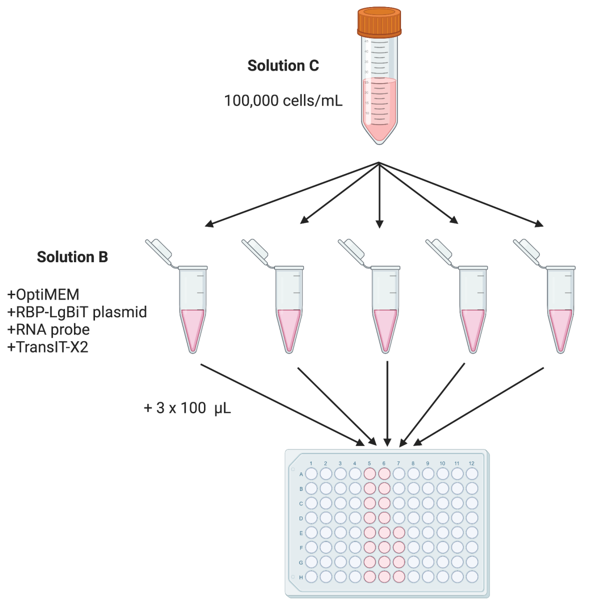

Figure S2. TransIT-X2® transfection workflow.

D. CellTiter-Glo® Assay

Cells were transfected following the Lipofectamine™ RNAiMAX or TransIT-X2® protocol and incubated in a white-bottom, tissue culture-treated 96-well plate (Corning cat #3917) for 24 h in a humidified incubator (37 °C and 5% CO_2). At 24 h, media was removed and replaced with 70 µL Opti-MEM™ and 70 µL CellTiter-Glo® reagent reconstituted according to the manufacturer's protocol. The plate was incubated for 30 min then chemiluminescence signal was collected on a BioTek Cytation3 plate reader.

E. EMSA

Pre-miRNA probe (500 nM) and purified SmBiT-HT[2] (5 µM) were incubated in 10 µL phosphate buffer (pH 8.0) for 1 h at room temperature. The reaction was quenched with 10 µL 2X RNA loading dye (95% formamide, 0.02% SDS, 0.02% bromophenol blue, 0.01% xylene cyanol, 1 mM EDTA in H_2O) and ran on TBE-Urea gels (10% for 45 min at 200V or 15% for 60 min at 200V). Gels were imaged with ChemiDoc™ Imaging System (Biorad).

F. Supplemental Figures

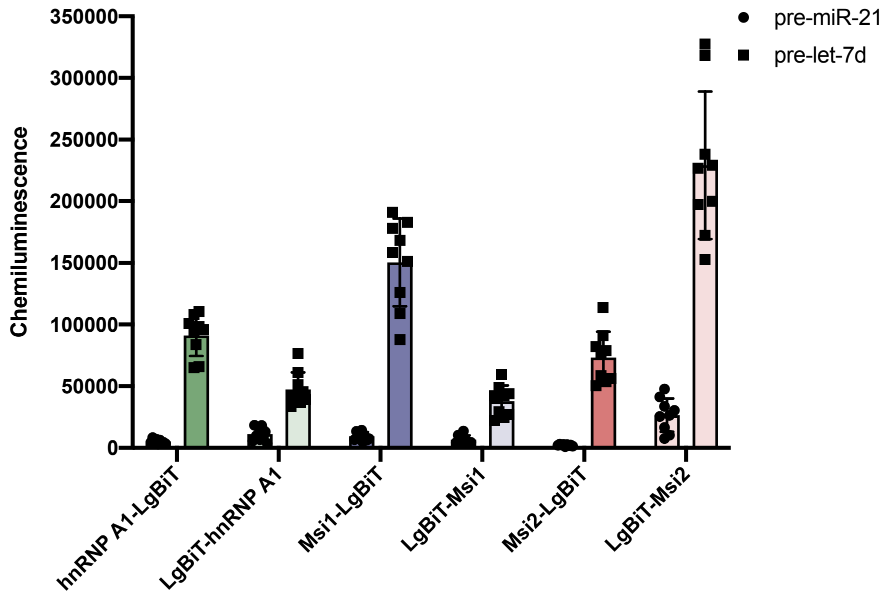

Figure S3. Raw chemiluminescence signal associated with Figure 3A.

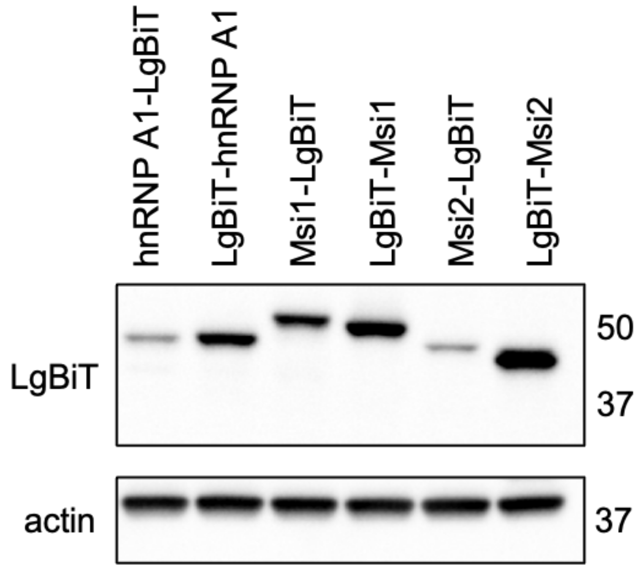

Figure S4. Expression of LgBiT-tagged RBPs in HEK 293 cells.

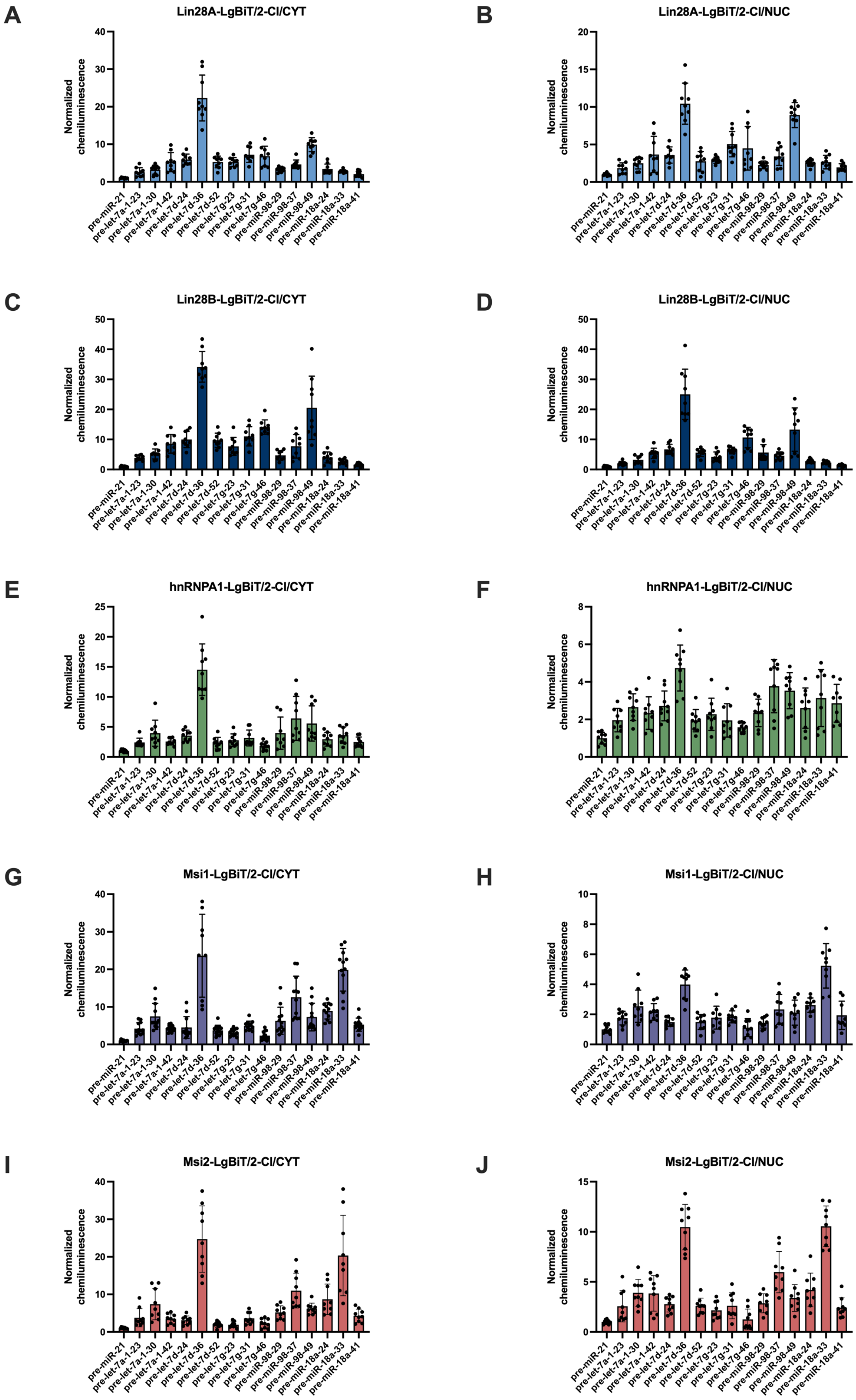

Figure S5. Normalized chemiluminescence from Figure 5.

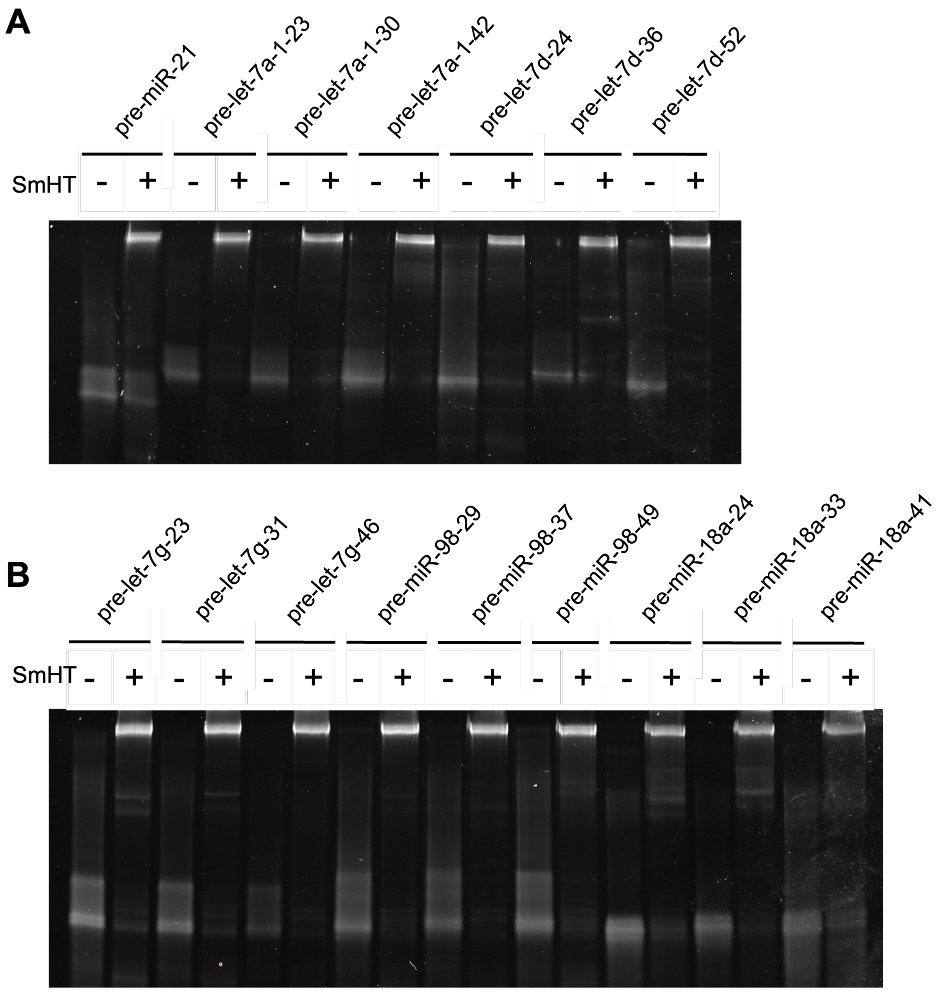

Figure S6. EMSA with the library of pre-miRNA probes and SmBiT-HT protein.

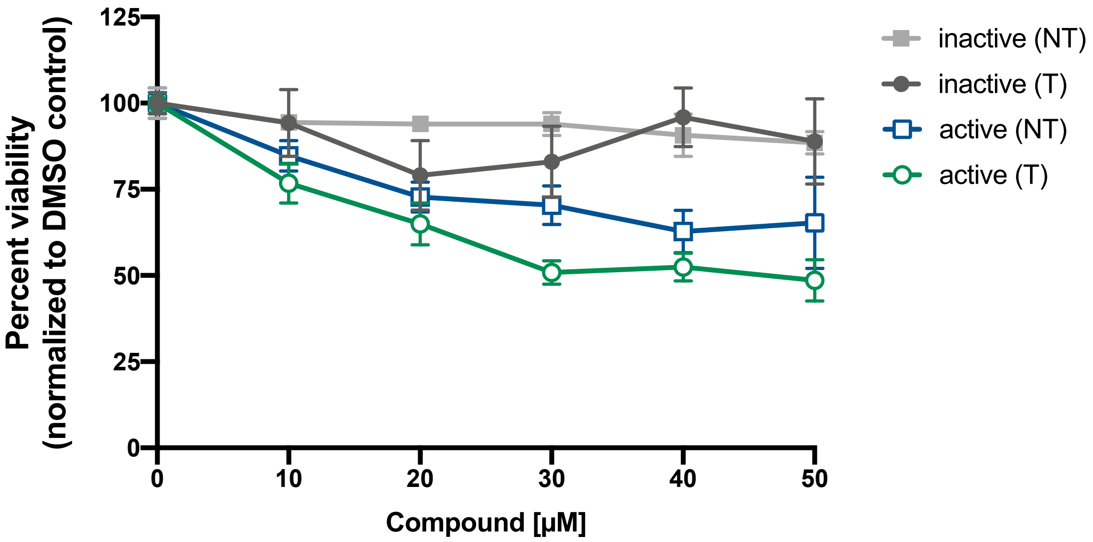

Figure S7. Cell viability of Flp-In 293 cells either not-transfected (NT) or transfected with Lipofectamine™ RNAiMAX and treated with either and inactive or active pre-let-7d/Lin28A inhibitor previously reported by the Garner Laboratory.[3]

G. References

[1] (a) S. L. Rosenblum, A. L. Garner, *Curr. Protoc.* **2022**, *2*, e358; (b) S. L. Rosenblum, D. A. Lorenz, A. L. Garner, *RSC Chem. Biol.* **2021**, 241-247.

[2] E. J. Sherman, D. A. Lorenz, A. L. Garner, *ACS Comb. Sci.* **2019**, *21*, 522-527.

[3] D. A. Lorenz, T. Kaur, S. A. Kerk, E. E. Gallagher, J. Sandoval, A. L. Garner, *ACS Med. Chem. Lett.* **2018**, *9*, 517-521.
